# Hypoxia Onset in Mesenchymal Stem Cell Spheroids: Monitoring With Hypoxia Reporter Cells

**DOI:** 10.3389/fbioe.2021.611837

**Published:** 2021-02-05

**Authors:** Carola Schmitz, Ekaterina Potekhina, Teresa Irianto, Vsevolod V. Belousov, Antonina Lavrentieva

**Affiliations:** ^1^Institute of Technical Chemistry, Gottfried Wilhelm Leibniz University Hannover, Hanover, Germany; ^2^Department of Metabolism and Redox Biology, Shemyakin-Ovchinnikov Institute of Bioorganic Chemistry, Moscow, Russia; ^3^Center for Precision Genome Editing and Genetic Technologies for Biomedicine, Pirogov Russian National Research Medical University, Moscow, Russia; ^4^Federal Center of Brain Research and Neurotechnologies, Federal Biomedical Agency, Moscow, Russia

**Keywords:** hypoxia sensor, cell spheroids, adipose tissue-derived mesenchymal stem cells, hypoxia reporter cells, oxygen concentration measurements

## Abstract

The therapeutic and differentiation potential of human mesenchymal stems cells (hMSCs) makes these cells a promising candidate for cellular therapies and tissue engineering. On the path of a successful medical application of hMSC, the cultivation of cells in a three-dimensional (3D) environment was a landmark for the transition from simple two-dimensional (2D) testing platforms to complex systems that mimic physiological *in vivo* conditions and can improve hMSC curative potential as well as survival after implantation. A 3D arrangement of cells can be mediated by scaffold materials where cells get entrapped in pores, or by the fabrication of spheroids, scaffold-free self-organized cell aggregates that express their own extracellular matrix. Independently from the cultivation method, cells expanded in 3D experience an inhomogeneous microenvironment. Many gradients in nutrient supply, oxygen supply, and waste disposal from one hand mimic *in vivo* microenvironment, but also put every cell in the 3D construct in a different context. Since oxygen concentration in spheroids is compromised in a size-dependent manner, it is crucial to have a closer insight on the thresholds of hypoxic response in such systems. In this work, we want to improve our understanding of oxygen availability and consequensing hypoxia onset in hMSC spheroids. Therefore, we utilized human adipose tissue-derived MSCs (hAD-MSCs) modified with a genetical sensor construct to reveal (I) the influence of spheroid production methods and (II) hMSCs cell number per spheroid to detect the onset of hypoxia in aggregates. We could demonstrate that not only higher cell numbers of MSCs, but also spheroid formation method plays a critical role in onset of hypoxia.

## Introduction

Human mesenchymal stem cells (hMSC) are extensively studied in the field of regenerative medicine. Their ability of self-renewal and differentiation makes them a promising tool for biomedical applications (Dominici et al., 2006), additionally hMSCs secrete cytokines and signal hormones which convey angiogenic and anti-apoptotic effects (Teixeira et al., 2013). Another promising bioregenerative potential of hMSCs comes from their ability to produce extracellular vesicles (EVs), which contain messengerRNAs, microRNAs, enzymes, signal proteins, etc. (Harrell et al., 2019) and support the regeneration and survival of damaged cells and tissues (Ophelders et al., 2016). When cultured on two-dimensional (2D) platforms, cells lack microenvironmental features that are present *in vivo*, like intensive physical and biochemical intercellular interactions, accumulation of signaling molecules or naturally occurring gradients of nutrients, oxygen and metabolic waste products. Researches try to mimic this complex interplay by the use of three-dimensional (3D) cell culture systems, that either require biocompatible or bioactive scaffolds like hydrogels (Caliari and Burdick, 2016; Kirsch et al., 2019) to allow a spatial arrangement of the cells, or through the fabrication of scaffold-free cellular aggregates—spheroids. In comparison to scaffold-based platforms, which contain an exogenic compounds, cells arranged as spheroids build their own extracellular matrix to maintain their 3D organization.

For 3D cell spheroid-based *in vitro* models, drug screenings and spheroid production for biomedical applications, robust and reproducible methods for spheroid fabrication are required. There are many techniques to produce cell spheroids: the traditional and historically the oldest *hanging drop* technique is cheap and easy (Foty, 2011) while *spheroid low attachment microplates* allow a more standardized fabrication procedure and better handling (Howes et al., 2014). In both cases the exact cell number per spheroid can be reproducibly set. Other techniques, including *liquid overlay* technique (Costa et al., 2018) or *spinner flask culture* (Lin and Chang, 2008) produce spheroids in large scale but lack the ability to exactly control spheroid sizes. Microfluidic systems, rotating wall vessel as well as magnetic levitation are more sophisticated, but require special equipment, which is not available to every laboratory (Ryu et al., 2019). One of the newest approaches for the large scale spheroid production are microstructured plates or cultivation chambers. Using these systems, hundreds of spheroids with same size can be generated (Dou et al., 2018).

Spheroid cultures were shown to be advantageous over traditional 2D cultivation since intercellular interactions are enhanced and cells built their own extracellular matrix similar to *in vivo*. During spheroid formation, cadherin and integrin glycoproteins mediate the construction of an adhesive network and take a significant role in cell signaling pathways (Weber et al., 2011). As a result of biochemical and mechanotransducive effects, hMSCs organized in a spheroid show increased angiogenic (Bhang et al., 2012; Cheng et al., 2013), anti-inflammatory (Bartosh et al., 2010; Murphy et al., 2017b) and immunomodulatory potential (Follin et al., 2016; Noronha et al., 2019), as well as increased stemness compared to cells cultivated in 2D (Lee et al., 2017). Moreover, increased cell survival of spheroids applied *in vivo* was observed by several research groups (Liu et al., 2013; Xu et al., 2016). Amos et al. (2010) applied adipose tissue-derived hMSCs spheroids in mouse models for dermal wound treatment and could show, that the injection of suspension cells was less successful than 3D cultivated and applied cells. It is important to note that cultivation in 3D aggregates allows MSC expansion under serum-free conditions (Alimperti et al., 2014). Moreover, even if cells were cultivated in 2D in several passages and then brought to the 3D spheroid cultures, their regenerative potential was significantly improved (Cheng et al., 2013). Application of 3D spheroid culture as priming strategy for enhanced therapeutic potential of MSCs is also described by Kouroupis et al. (2019). Thus, numerous reviews on expansion methods of hMSCs indicate advantages of cultivation in 3D spheroids prior implantation (Egger et al., 2018; Mastrolia et al., 2019; Noronha et al., 2019; Lavrentieva et al., 2020).

Besides MSCs expansion for clinical applications, spheroids created from other cell types provide a suitable platform for studies of tumor growth and behavior, enabling researches to investigate the influence of the microenvironment on 3D arranged cells similar to tumors *in vivo* (Gilkes et al., 2014). Many researchers agreed, that cell metabolism and oxygen consumption in these 3D aggregates lead to the formation of a hypoxic core, which is closely related to altered cell response toward many tumor treatments compared to studies performed in 2D (Däster et al., 2017; Nunes et al., 2019). While application or onset of hypoxia in tumor spheroids is intensively studied, little is known about presence of hypoxia in MSCs aggregates. Tumor spheroids where shown to build a necrotic or hypoxic core (Khaitan et al., 2006; Riffle and Hegde, 2017), in contrast, hMSC spheroids seem to adapt to 3D cultivation by decreasing their packaging density and therefore enable easier oxygen diffusion (Murphy et al., 2017a). Due to different spheroid formation platforms, spheroid sizes and experimental setups, the formation of a hypoxic core and oxygen availability in non-tumor MSCs spheroids remains unclear.

Since cell response to hypoxia is mainly mediated by the stabilization of hypoxia inducible factor 1α (HIF-1α) (Majmundar et al., 2010), this key regulator serves as a great tool to detect and investigate hypoxic conditions *in vitro* and *in vivo*. Under hypoxic conditions, the protein degradation of the constitutively expressed HIF-1α is inhibited (Maxwell et al., 1999) and HIF-1α can enter the cell core, dimerise with HIF-1β and bind to the hypoxia responsive elements (Jiang et al., 1996), triggering oxygen-depended regulation of over 300 genes (Mole et al., 2009). Erapaneedi et al. (2016) utilized HIF-1α stabilization and hypoxia responsive elements to generate a genetic construct which modulates reporter protein expression, when cells are exposed to hypoxia. The system relies on the expression of fluorescent UnaG protein (Kumagai et al., 2013), whose maturation process is independent from presence of molecular oxygen. While this construct provides a great tool for hypoxia research in general, the application of hypoxia reporter cells is invaluable for 3D cell culture applications (Schmitz et al., 2020). Finally, a non-invasive hypoxia monitoring allows the live analysis of the hypoxic state of single cells within complex constructs.

In the present study, we utilized human adipose tissue-derived MSCs (hAD-MSCs) modified with genetical sensor construct to reveal (I) the influence of spheroid production methods and (II) MSCs cell number per spheroid to detect the onset of hypoxia in aggregates. Additionally, we performed oxygen concentration measurements in fabricated spheroids. We could demonstrate that not only higher cell numbers of MSCs, but also spheroid formation method plays a crucial role in onset of hypoxia.

## Materials and Methods

### Reporter hAD-MSCs

Hypoxia reporter MSCs were created as described earlier (Schmitz et al., 2020). Briefly, hAD-MSC were isolated from adipose tissue after abdominoplasty applying the protocol of Zhu et al. (2008). All patients provided their informed consent, as approved by the Institutional Review Board (Hannover Medical School) with the reference number 3475-2017. The obtained cells were characterized by MSC-typical surface markers and functional properties (Dominici et al., 2006) and the presence of adipogenic, chondrogenic, and ostogenic differentation potential. hAD-MSCs were used for lentiviral transduction, to stably integrate HRE-dUnaG (Erapaneedi et al., 2016) sequence. Efficient transduction with HRE-dUnaG and oxygen-dependend UnaG expression was shown in our previous work (Schmitz et al., 2020). Surface antigen expression of hypoxia reporter modified MSCs was evaluated by flow cytometry. Cells cultivated in 2D were harvested by accutase treatment, washed with cold blocking buffer (2% human serum in PBS) and resuspended in 100 μl of cold washing buffer to a concentration of 10 × 10^4^ cell/100 μl. Each cell solution was incubated for 20 min in the dark at room temperature with respective antibodies. Cells were tested positively for MSC specific antigens as CD73 (ecto-5′-nucleotidase) and CD105 (endoglin). Reporter MSCs were tested negatively for non MSC specific antigens CD34, CD45 and CD31 (Supplementary Figure 1). Antibodies with respective reporter dye and isotype controls: PE Mouse IgG1, κ Isotype Control was compared with PE Mouse Anti-Human CD73 and PE Mouse anti-Human CD105. FITC Mouse IgG1, κ Isotype Control was used for APC Mouse Anti-Human CD34 and FITC Mouse Anti-Human CD45 testing. PE-CF594 Mouse Anti-Human CD31 and PE-CF594 Mouse IgG1, κ Isotype were applied (all purchased from BD Bioscience, USA). Cells were cultivated in α-minimum essential medium (MEM; Thermo Fisher Scientific, USA) containing 1 g/L glucose, 2 mM l-glutamine, 10% human serum, and 50 μg/ml gentamicin (Merck KGaA) in a humidified incubator (C16, Labotect, Germany) at 37°C at 5% CO_2_.

### Spheroid Fabrication, Imaging, and Spheroid Diameter Analysis

Cells (passage 8–10) expanded in 2D were harvested by accutase treatment. Spheroids of different cell numbers (1, 3, 7.5, 15, 30 × 10^4^ cells) were formed using different fabrication methods. In the first technique, cells were seeded into 96-well Corning® Costar® ultra-low attachment plates in 150 μl of αMEM supplemented with 10% human serum and 0.5% gentamycin. For the hanging drop spheroid fabrication, drops of cell suspensions (30 μl) were seeded into Terasaki microassay plate wells (Greiner Bio-One, Austria). For spheroid formation in hanging drop, the plate containing the cell suspensions was turned upside down and placed on the lid. To prevent fluid evaporation 2–3 ml PBS were previously pipetted in the lid. For both methods spheroid formation time was 24 h under similar culturing conditions as mentioned above. After 24 h spheroids were transferred to 24 well lumox multiwell plates (Sarstedt, Germany) and images of at least three spheroids were taken by Cytation®5 multimode imaging reader (BioTek® Instruments, USA). To determine the spheroid diameter, the average of the length of the x-axis and the y-axis of each spheroid was measured in paint.net, using the microscopic scale to translate the length in pixel into decimal system.

### Kinetic Imaging of Spheroid Fabrication

The spheroid formation process and onset of hypoxic response during formation were monitored by kinetic imaging via Cytation®5 multimode imaging reader for low attachment plates and with incubator microscope (Lumascope 600, Etaluma Inc., Carlsbad, CA, USA) for Terasaki plates and microstructured plates. For low attachment plates, magnification of 4x was used to take one bright field channel (LED intensity: 5, exposure time: 100, gain: 0.6) and one GFP channel (LED intensity: 6, exposure time: 100, gain: 0) image every 10 min for 24 h. The images were used to create videos by the integrated Gen.2.08 software. For Terasaki plates and microstructured plates, images were taken with the help of Lumaview (Etaluma Inc., Carlsbad, CA, USA) software in bright field and GFP channels every 1 min and videos were created using VideoDub software.

### Spheroid Dissociation and Flow Cytometric Analysis

For the flow cytometric analysis, spheroids were washed with PBS, transferred into 2 ml reaction tubes and 200 μl TrypLE Express solution (Thermo fisher, Germany) was added for enzymatic digestion. Cells were incubated at 37°C. Additionally, every 10 min cell separation was stimulated by mechanical disruption through rough pipetting using a glass pipette. After 20 min of incubation, another 200 μl TrypLE Express solution was added to the reaction tube. This process was performed until up to 1 h, or ended earlier, if cells were already singularized. The cells were centrifuged (5 min, 200xg) and the pellet resuspended in PBS for flow cytometric analysis. The flow cytometer BD FACSAria™ Fusion (Becton Dickinson, USA) was used to evaluate single cell fluorescence. The target cell population was excited at 488 nm and fluorescence detected via FL 1 detector (533/30 nm) to analyze the cellular expression of UnaG protein.

### Large Scale Spheroid Formation and Imaging in Microstructured Plates

The third studied spheroid fabrication technique was microstructured plates (Sphericalplate 5D, Kugelmeiers Ltd., Switzerland). Here, spheroids with two different cell numbers (~270 cells and 1,300 cells) were created and studied. Therefore, either 0.2 × 10^6^ or 1 × 10^6^ cells were plated into the microstructured plate in 1 ml cell culture medium accordingly to the manufacturer's instructions. To avoid air bubbles formation, plates were pre-incubated with warm PBS for 2 h prior cell seeding.

### *In situ* Oxygen Measurements

For the measurement of local oxygen concentrations in spheroids OPAL Optical O_2_ Measurement System (Colibri Photonics GmbH, Germany) was used. The OPAL system, integrated into the fluorescent microscope, allows quantitative optical non-invasive oxygen measurements in 2D and 3D cell cultures. During spheroid formation, 0.4 μl CPOx-orange microbeads (50 μm polystyrene beads, 5 mg/ml suspension in PBS) were added in each well of low attachment plate or to each drop of Terasaki plate, resulting in 4–15 beads per spheroid. For the performed oxygen measurements, the analysis was always focused on the center of the 3D aggregate. If several beads were incorporated in the spheroid, the system evaluated an average oxygen tension for all beads in the microscopes focus area. For microstructured plates, microbeads were distributed evenly in the microwells, before cell suspension for spheroid formation was added. After spheroid formation, oxygen measurements were performed at room temperature at Ex530nm/Em600nm using OPAL system. Three spheroids per platform and per cell number were measured to obtain mean values.

### Data Analysis

Data is shown as mean value of at least 3 measurements. The error bars show the positive/negative standard deviation. Statistical significance was assessed with the one way ANOVA (Microsoft Exel, Microsoft corporation, USA).

## Results

**Figure 1:**
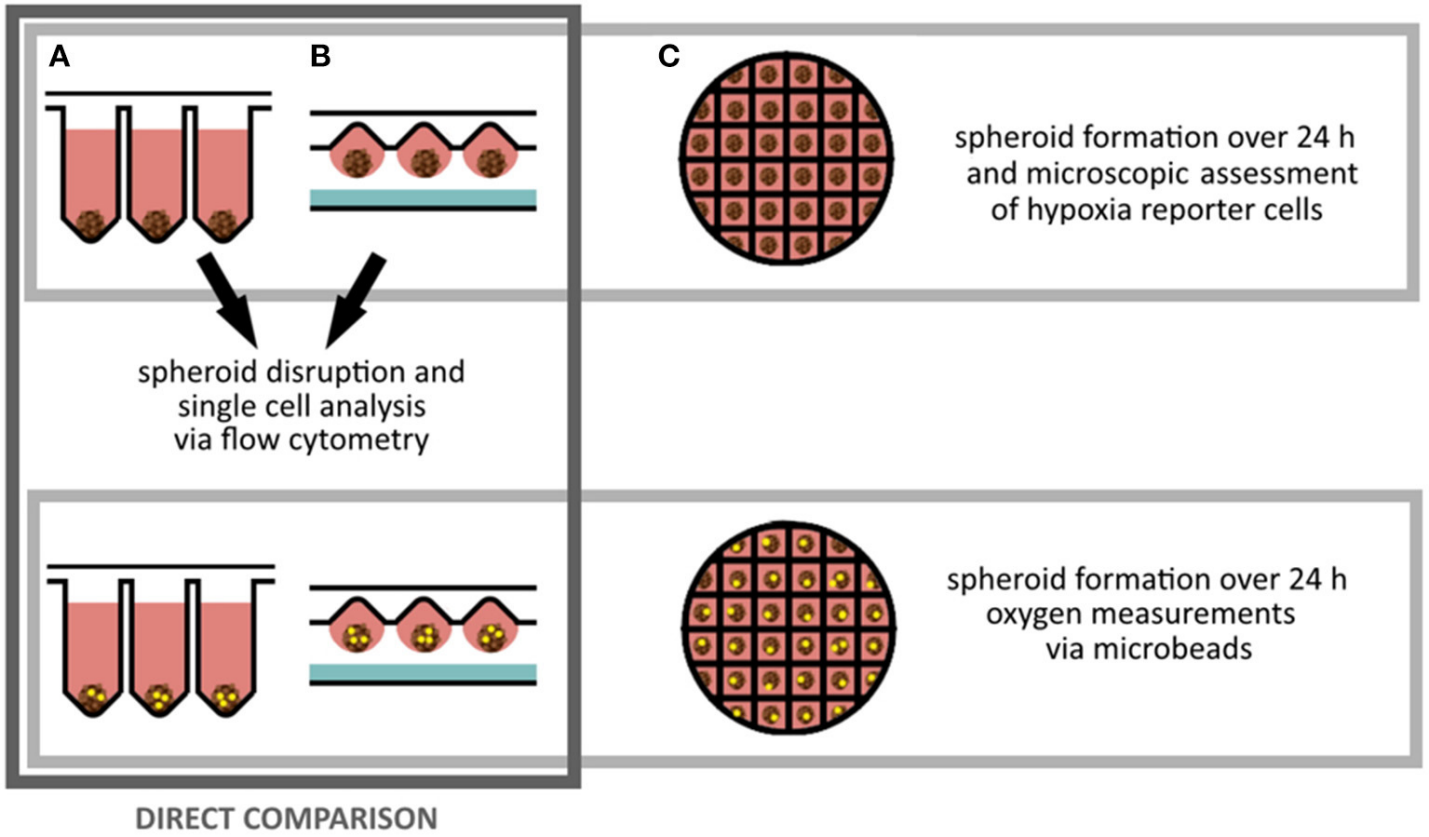
Experimental planning: Spheroids of MSC reporter cells were fabricated for 24 h on three different platforms. **(A)** Ultra low attachment plates, **(B)** by hanging drop method, **(C)** on microstructured plates. Spheroids created on ultra low attachment plates and by hanging drop method were singularized for flow cytometry analysis to investigate the sensor cell output. For all methods oxygen sensitive microbeads were applied to determine local oxygen concentrations inside of the spheroids.

Three different spheroid fabrication platforms (ultra low attachment plate, hanging drop and microstructured plates) were applied to obtain spheroids of different sizes. A direct comparison between ultra low attachment plates und hanging drop method was performed in terms of fluorescent hypoxia reporter protein expression (by fluorescent microscopy and flow cytometry). Additionally, *in situ* oxygen measurements were executed for spheroids of different sizes and fabrication platforms. For microstructured plates, fluorescent microscopy and local oxygen measurements in cell culture medium near spheroids was performed (Figure 1).

### Spheroid Fabrication in Low Attachment Plates and by Hanging Drop Method

**Figure 2:**
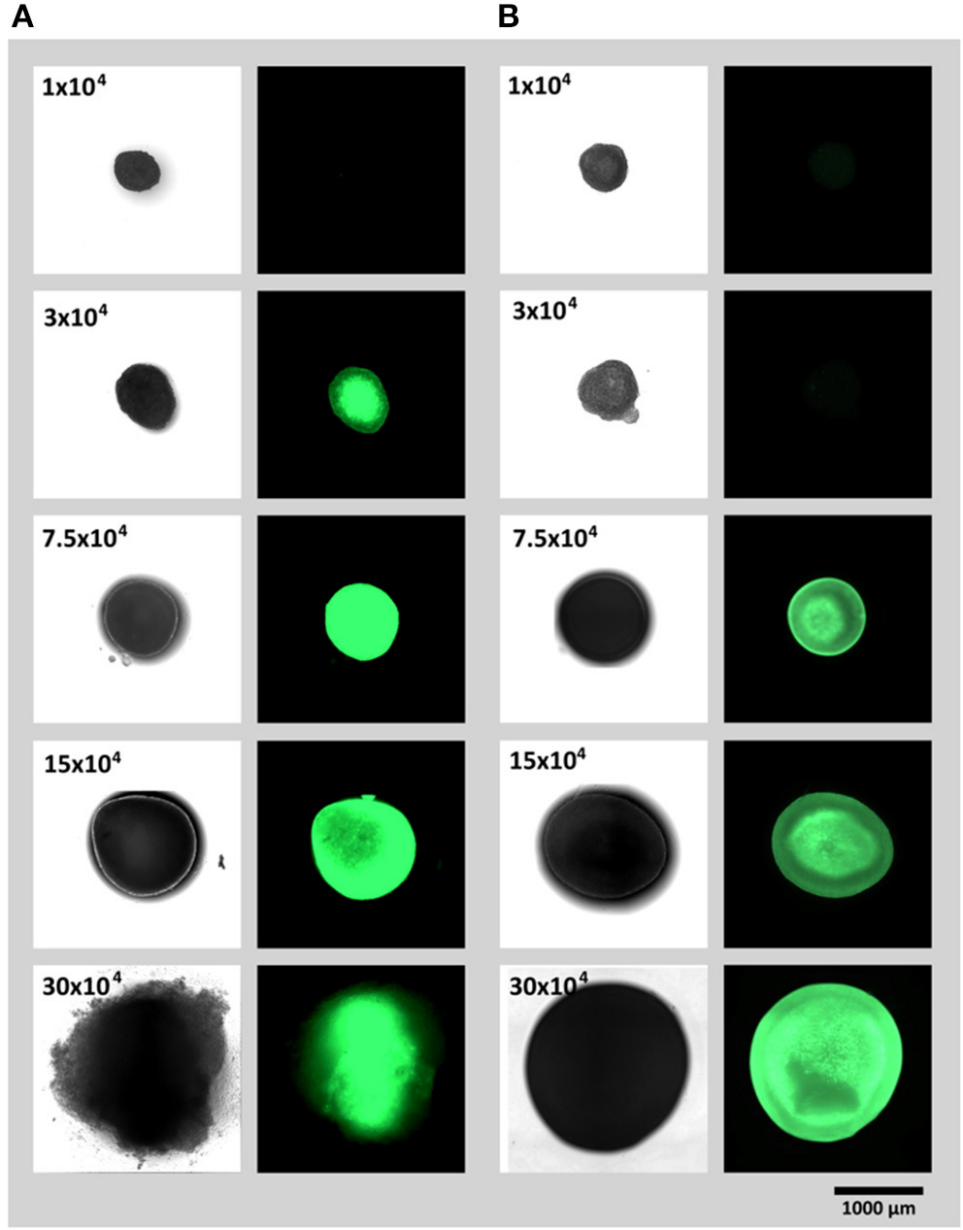
Spheroid formation and resulting reporter hAD-MCSs fluorescence (UnaG expression) after 24 h on **(A)** ultra low attachment plates and **(B)** via hanging drop method.

To study (1) the influence of spheroid cell number and (2) spheroid fabrication platform on the onset of hypoxia in 3D cellular aggregates, spheroids of similar cell numbers (1, 3, 7.5, 15 or 30 × 10^4^ cells) were created in ultra low attachment plates or via hanging drop method. To lower the variance of spheroid shape due to different drop geometries, Terasaki plates were used for creation of hanging drops. Spheroids fabricated on both platforms were evaluated after 24 h (Figures 2A,B). For both, the ultra low attachment plate and the hanging drop method, expression of UnaG protein as a hypoxia reporter protein could clearly be monitored. For the spheroid formation in well plates, a strong signal was obtained from a spheroids of 3 × 10^4^ cells and larger (Figure 2A), for the hanging drop method the reporter protein expression starts at spheroid size from 7.5 × 10^4^ and larger (Figure 2B). Moreover, microscopically detected UnaG fluorescence was not only detected in smaller spheroids in the low attachment plates, but this fluorescence was remarkably higher in the spheroids of the same size than in the ones created with hanging drops. Hypoxic core was seen in spheroids with 3 × 10^4^ cells in the low attachment plates and in spheroids with 7.5 × 10^4^ and 15 × 10^4^ cells in hanging drop plates. In low attachment plates, fluoresce of reporter cells was spheroids was even distributed all over the aggregates with 7.5 × 10^4^ cells and larger. The largest tested spheroids (30 × 10^4^ cells) demonstrated decrease of fluorescence and spheroid dissociation in ultra low attachment plates. Spheroids of the same size in the hanging drop plate demonstrated high fluorescence intensity, but retained integrity.

### Kinetic Imaging of Spheroid Formation

The spheroid formation process and onset of sensor fluorescence was also monitored via kinetic imaging. Time-lapse videos are presented in the Supplementary Videos 1, 2. For both fabrication platforms, first signals of UnaG expression could be monitored 7 h after start of spheroid formation. Due to the different plate geometries, two different imaging platforms had to be used, which allowed detection of fluorescence but could not be directly compared in terms of fluorescence assessment.

### Spheroid Dissociation and Flow Cytometric Analysis

**Figure 3:**
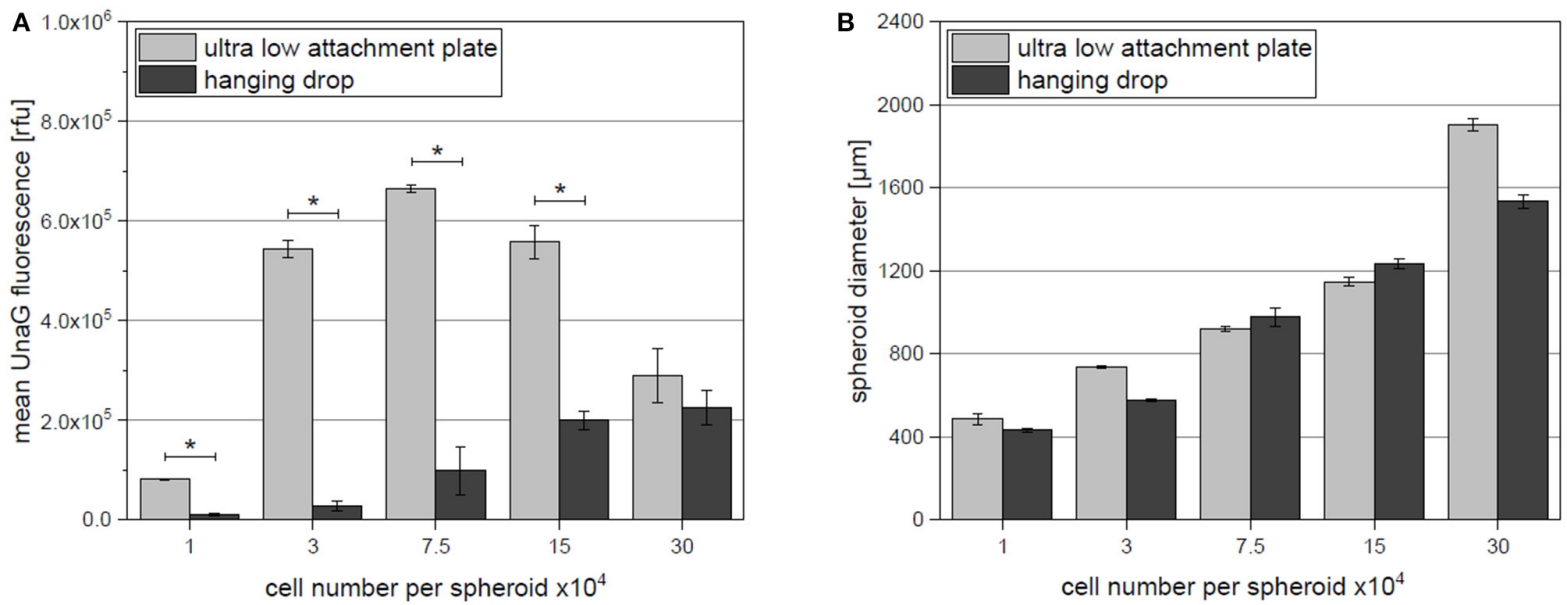
**(A)** Mean single cell fluorescence determined by flow cytometry. For both fabrication platforms (ultra low attachment plate and hanging drop) hypoxia reporter hAD-MSCs were cultivated as spheroids and dissociated to facilitate single cell analysis. **(B)** Respective spheroid diameters as obtained by both fabrication platforms. Data represent the mean ± SD for a 3-fold determination. *p < 0.05.

For quantitative analysis of reporter cell fluorescence, flow cytometry was additionally performed. Spheroids were dissociated after 24 h of fabrication to allow access to single cell analysis. The average cell fluorescence for each spheroid size and the respective method is displayed in the Figure 3A. With the help of highly sensitive flow cytometry, increase of fluorescence in the low attachment plates (in comparison to hanging drops) was already detected in the smallest studied spheroids (1 × 10^4^ cells per spheroid). Supporting the results of fluorescent microscopy significantly increased mean UnaG fluorescence was detected in aggregates formed in ultra low attachment plates if compared to hanging drop plates. For ultra low attachment plates, highest detected fluorescence was in the spheroids with 7.5 × 10^4^ cells. For larger spheroids the mean fluorescence was decreasing. In contrast, the mean fluorescence of spheroids created by hanging drop method was much weaker, compared to the ultra low attachment plate. Moreover, a constant increase in mean UnaG fluorescence with increasing spheroid size could be detected.

To additionally study the influence of the different fabrication platforms on spheroid size, which affect oxygen diffusion inside of the 3D construct, the diameters of the undissociated spheroids were evaluated (Figure 3B). For most sizes, spheroid diameter appears to be similar for both methods. For 30 × 10^4^ cells per spheroid, the average diameter appears to be larger, but when compared to the image in Figure 2A and the Video (Supplementary Video 1), the actual spheroid seems to dissociate causing the increase in the measured diameter.

### *In situ* Oxygen Measurements in Cell Spheroids With Oxygen Sensitive Microbeads

**Figure 4:**
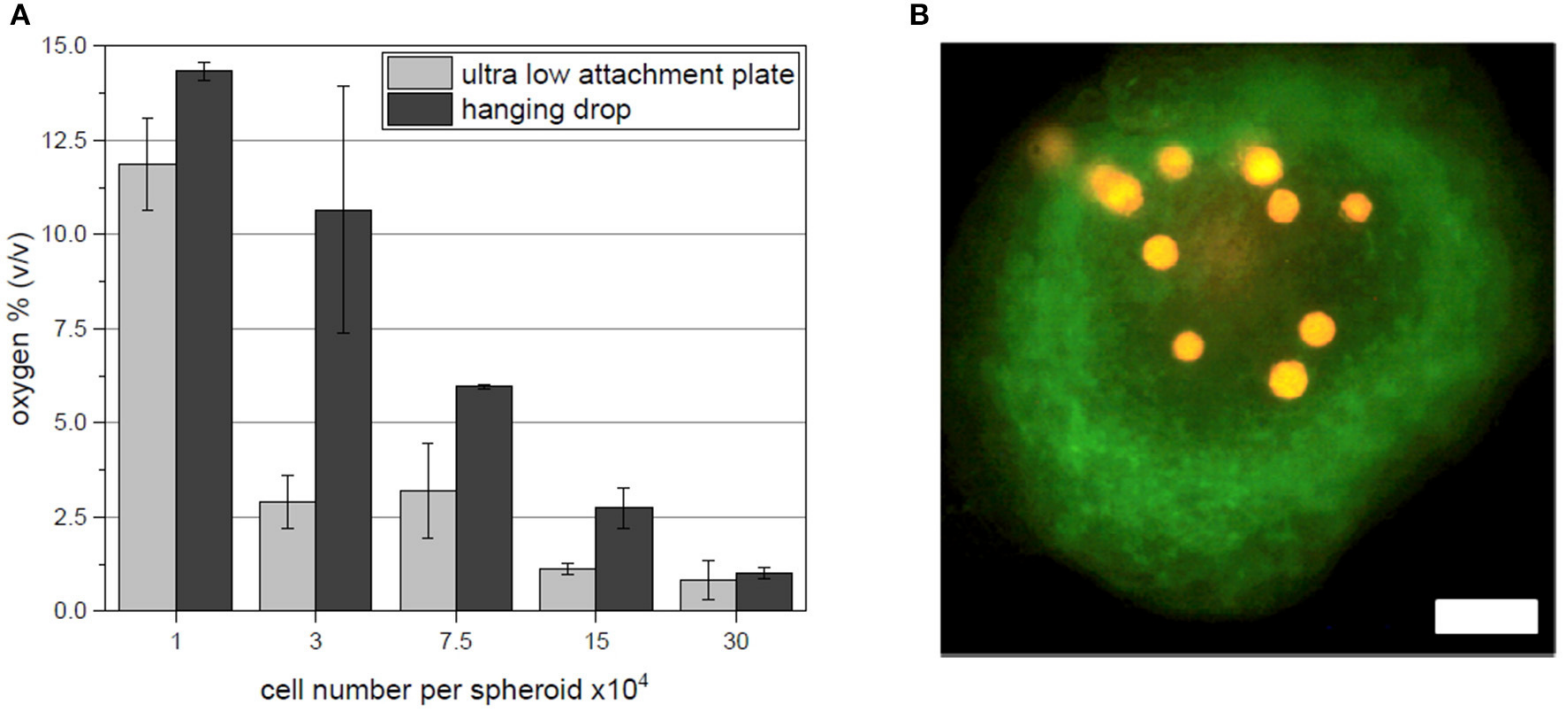
**(A)** *In situ* oxygen measurements for each spheroids size and fabrication platform. **(B)** Exemplary fluorescent image of microbeads (orange) incorporated in a spheroid of 7.5 × 10^4^ hypoxia reporter hAD-MSC (green) fabricated in ultra low attachment plates. Scale bar: 200 μm.

In addition to the acquired fluorescent hypoxia signal output evaluation, oxygen sensitive microbeads (CpoX orange) were incorporated in spheroids of each size and fabrication platform. Evaluation of the local oxygen concentrations was consistent with fluorescent reporter cell output. With increasing spheroid size, the oxygen content in the 3D cell aggregate drops (Figure 4A). Supporting flow cytometry results, spheroids fabricated by hanging drop method show higher local oxygen tensions as spheroids created in ultra low attachment plates. Spheroids of 30 × 10^4^ cells reach oxygen concentrations as little as <1% (v/v). Important to note that despite this system allows a local oxygen evaluation also for 3D cellular constructs, the spatial positioning of the microbeads can hardly be controlled (Figure 4B).

### Application of Hypoxia Reporter Cells in Large Scale Spheroid Formation in Microstructured Plates

**Figure 5:**
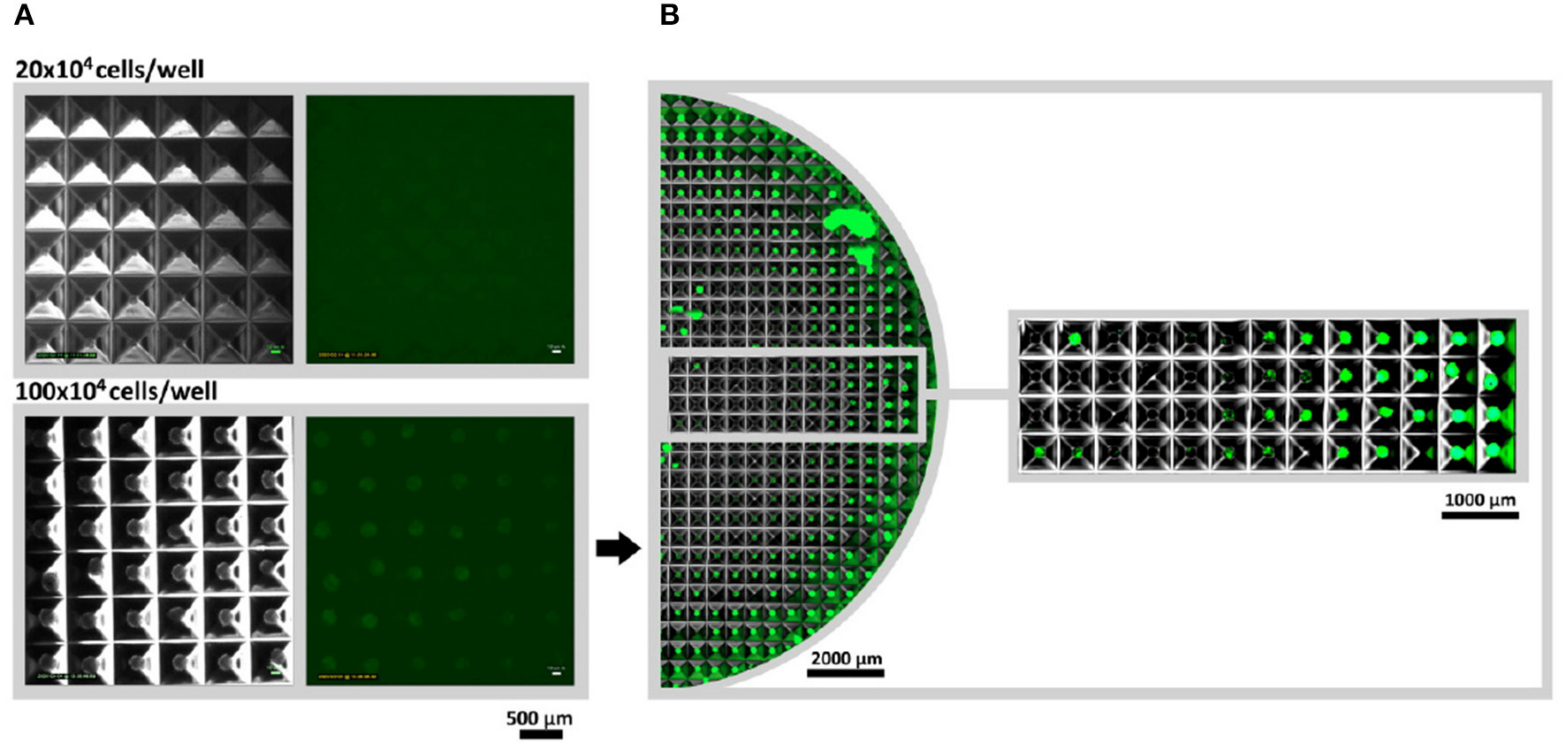
Large scale spheroid formation in microstructured wells. **(A)** Spheroids were monitored via Lumascope for 20 × 10^4^ and 100 × 10^4^ cells per well after 24 h of formation time. Larger spheroids (100 × 10^4^ cells/multiwell) would express the hypoxia reporter protein UnaG, while low numbers (20 × 10^4^ cells/multiwell) did not show a signal. For both studied seeded cell numbers images were taken from the outer corner of the well to guarantee comparability. **(B)** For 100 × 10^4^ cell/multiwell half the well was accessed via image stitching. Time-laps video of spheroid formation and onset of fluorescence can be seen in Supplementary Video 3.

According to the manufacturers, microstructured plates allow fast creation of large amounts of spheroids with controlled diameter and identical biological properties. Hypoxia reporter cells were applied in a microstructured plate to control the stabilization of HIF-1α and to confirm the uniformity of large scale spheroid formation. Cells were seeded in 2 different densities: 20 × 10^4^ cells per well and 100 × 10^4^ cells per well and incubated for 24 h. While no fluorescent signal could be monitored at 20 × 10^4^ cell/well (~267 cells per spheroid, spheroid diameter of 100 μm), 100 × 10^4^ cells/ well (~1,333 cells per spheroid, spheroid diameter of 180 μm) led to the hypoxia reporter signal (expression of UnaG protein) (Figure 5A). Interestingly, a gradient of fluorescence could be detected over the microstructured well, when the entire well was accessed. Spheroids at the outer edge of the microstructured well-expressed gradually more UnaG protein as spheroids formed in the center (Figure 5B). Big chunks of cells appearing in the image are a result of plate transportation to the imaging system. Due to the agitation, spheroids leave their wells and unwontedly accumulate with other spheroids. Time laps microscopy, however clearly demonstrated the absence of fluorescent spheroids in the middle of the well (Supplementary Video 3).

### Oxygen Measurement in Medium in Microstructured Wells

**Figure 6:**
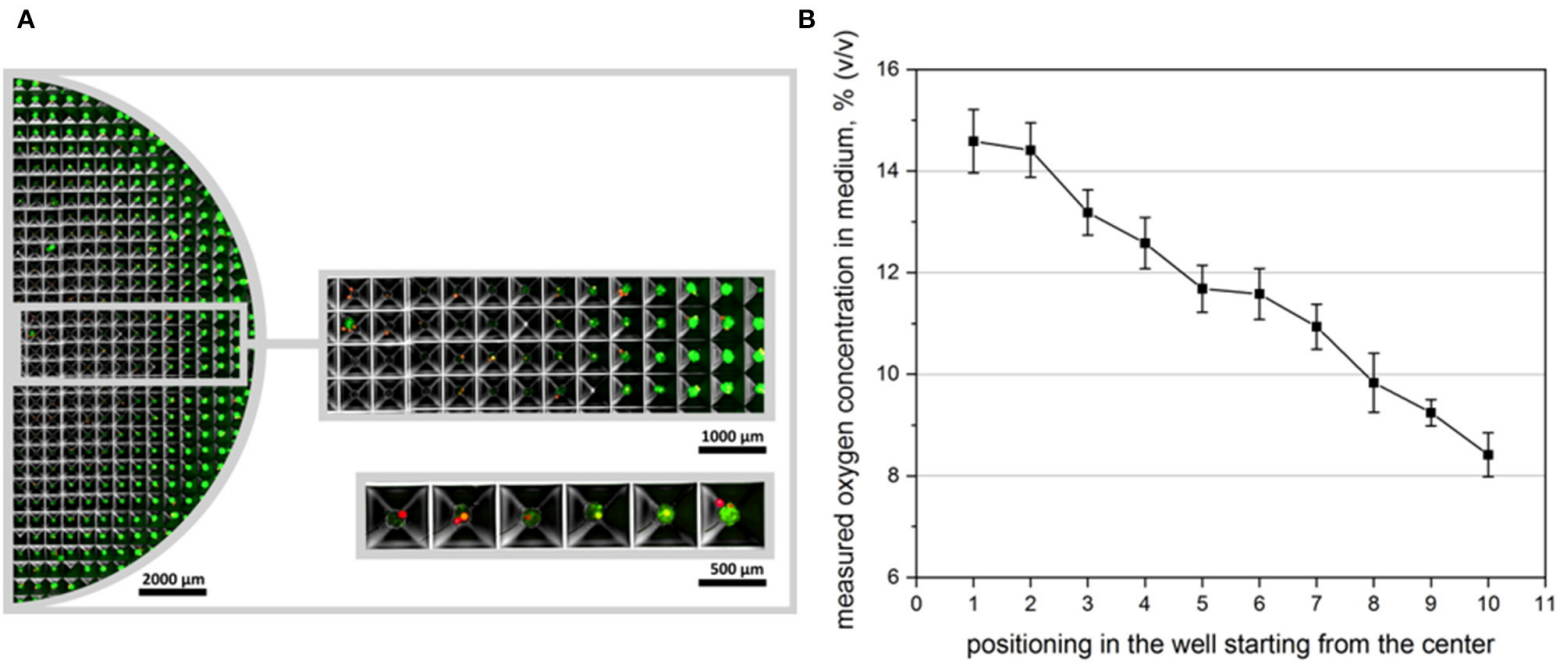
Oxygen measurement in microstructured wells. **(A)** Overview over half the microstructured well with seeded oxygen sensitive microbeads. CpoX beads (red) were placed in the microwells previously to reporter cell seeding. 100 × 10^4^ cells/multiwell are displayed 24 h after cell seeding. **(B)** Accordingly oxygen concentrations in different microwells were measured. Position 1 represents microwells mostly in the center, while position 10 represents wells at the outer edge of the well. Measurements were taken in even distances in dependence of the readability of the applied microbeads. A clear gradient in oxygen availability could be monitored as confirmed by reporter cell output.

Oxygen sensitive microbeads were applied in microstructured plates to measure the local oxygen content of the cell culture medium when spheroids were formed using 100 × 10^4^ cells per well. In contrast to previous oxygen measurement experiments, microbeads could not be embedded inside of spheroids since microbead diameter was too large (50 μm) to be incorporated in small (180 μm) spheroids. Here, microbeads were either positioned at the edge of the spheroid, or underneath the 3D cell aggregate in medium (Figure 6A). The resulting oxygen concentrations were evaluated with OPAL system, starting in the center of the microstructured well (position 1) to the outer edge (position 10). A clear gradient in oxygen tension in medium could be monitored which confirmed reporter cells output (Figure 6B).

## Discussion

MSCs isolated from different tissues considered to be promising candidates for cell therapies and tissue engineering. In most of the cases, after isolation, MSCs must be expanded *in vitro* prior injection or application in tissue engineered constructs. Cultivation of MSCs in 3D cellular aggregates (spheroids) helps to preserve their biological functions and increase their therapeutic potential (Murphy et al., 2017a; Lavrentieva et al., 2020). Cultivation in spheroids enhances secretion of MSCs angiogenic, immunomodulatory, pro- and anti-inflammatory factors, as well as their differentiation potential (Cesarz and Tamama, 2016). Even if only shortly cultivated in 3D spheroids, MSCs demonstrate improved biological functions.

The reasons of higher MSCs curative properties after cultivation in 3D spheroids seems to be an increase of pluripotent gene expression via relaxation of cytoskeleton tension (Zhou et al., 2017), lower vascular obstruction via small cell size preservation (Ge et al., 2014) and increased expression and secretion of rescue factors as a sequence of hypoxia (Potapova et al., 2007).

There are several methods for *in vitro* spheroid formation, including centrifugation, low attachment plates, hanging drops and structured microwells (Bartosh et al., 2010; Egger et al., 2018; Lavrentieva et al., 2020). Each method has advantages and disadvantages and is usually chosen according the subsequent application or experimental set-up. With the help of hypoxia reporter cells we for the first time directly detected the onset of hypoxia in spheroids of different diameters and compared the influence on different spheroid fabrication platforms. Two fabrication platforms with different features were directly compared: (1) ultra low attachment microplates, were cells accumulate on the bottom of the wells and oxygen is mostly available through diffusion throughout the medium overlay, and (2) hanging drop in Terasaki plates, where the spheroid accumulates in the drop and the cells are almost directly exposed to the surrounded oxygen-rich atmosphere. Additionally, we applied the hypoxia reporter cells on microstructured wells for large scale spheroid formation and could demonstrate unequal hypoxia reporter protein expression if higher cell numbers were used. In the first part of our study we manufactured spheroids of 1 × 10^4^, 3 × 10^4^, 7.5 × 10^4^, 15 × 10^4^, and 30 × 10^4^ hAD-MSCs in spheroid microplates and via hanging drops in Terasaki plates. For large cell numbers (>7.5 × 10^4^ cells per spheroid) strong hypoxia reporter protein expression could be monitored. Interestingly, for 3 × 10^4^ cells per spheroid fluorescent hypoxia signal was present when 3D aggregates were formed in ultra low attachment plates, but no microscopically visible fluorescence was detected for spheroids created via hanging drop method. One possible explanation could be the difference in culture medium volumes used in both methods. A maximal possible medium drop volume for hanging drop method was 30 μl (to avoid the fall of the drop), compared to 150 μl media used for ultra low attachment plates. Additionally, to the larger gas-liquid interface in hanging drops, small drop volume seems to allow a better oxygen supply by decreased oxygen diffusion distances. Disadvantageously, small media volumes can lead to the fast drop of nutrients, so ultra low attachment plates, which facilitate spheroid formation in higher medium volumes, are widely used in scientific community. However, application of hypoxia reporter MSCs demonstrated that in the ultra low attachment plates oxygen transport limitation through cell culture media can cause an earlier onset of hypoxia if compared to hanging drops. These findings were also supported by further flow cytometric analysis.

The spheroids of each fabrication method were dissociated by proteases and fluorescent reporter protein expression was quantified by flow cytometric analysis. We were able to spot a significant increase in fluorescent signal output when ultra low attachment microplates were used. The strongest difference was detected for spheroids of the size of 3 × 10^4^ and 7.5 × 10^4^ cells. Unexpectedly the mean fluorescence of spheroids of 15 × 10^4^ and 30 × 10^4^ cells decreases in ultra low attachment microplates. As shown in Figure 1A the spheroid of 30 × 10^4^ cells has loose edges. When watched in the respective formation video (Supplementary Video 1), the spheroid fist forms and then cells start to dissociate. This indicates that cells of the two largest spheroids (15 × 10^4^ and 30 × 10^4^) possibly die due to strong oxygen deprivation, which in turn could explain the decrease in fluorescence.

The limitation of the flow cytometric analysis is the breakdown of spatial spheroid organization by dissociation. When the spheroids get dissociated for analysis, cells from the center of the spheroid, where strongest hypoxia is expected, and cells of the outer areas, where oxygen transport limitation are supposed to be weak, get mixed up. As a result, only mean fluorescence of the entire spheroid population can be obtained. As shown in our earlier works, the fluorescent signal of the hypoxia reporter hAD-MSCs at 2.5% O_2_ in 2D cultures reached an average signal of ~1.3 × 10^6^ RFU (Schmitz et al., 2020). The detected average signal in this work is twice weaker (max. mean fluorescence of 6.6 × 10^5^ RFU). In contrast, if looking on the highest fluorescent 1% of the cell population, the fluorescence signal intensity is similar to the highest cell fluorescence detected in 2D by 2.5% O_2_ for 24 h (data not shown). This indicates, that some of the cells in the studied spheroid cultures were exposed to oxygen concentrations as low as 2.5% O_2_. As confirmed by other researches (Groebe and Mueller-Klieser, 1991; Barisam et al., 2018) a gradient of oxygen is maintained within the spheroid, often causing hypoxia in the spheroid core (hypoxic core), while cells at the spheroid edge are not affected by oxygen diffusion limitations. Consequently, cells at the outer edge of the 3D aggregate experience less or even no oxygen deprivation and weaken the average signal intensity. Dead cells, which do not express UnaG could additionally weaken the average fluorescent signal.

The application of the OPAL system for *in situ* oxygen measurements confirms the findings of the previous results. Increasing spheroid sizes cause lower oxygen concentrations and ultra low attachment plates show fast drop of oxygen tension signals if compared to spheroids fabricated as hanging drops. While the used microbeads based system provides a great tool for oxygen measurements in 3D cell culture applications, the spatial incorporation of the beads could hardly be controlled and Z-axis positioning of beads could not be precisely evaluated. Nevertheless, performed measurements demonstrated the clear trend of stronger oxygen limitations in the ultra low attachment plate.

The application of different spheroid formation methods can influence the spheroid packaging density and consequently spheroid diameters. Evaluation of spheroid size demonstrated no difference between hanging drop and low attachment plate—fabricated spheroids (except for 30 × 10^4^, as discussed before). Thus, only spheroid fabrication platform and medium volume influence the onset of hypoxia. For example, we found that for spheroids of 7.5 × 10^4^ cells the average spheroid diameter is similar for both fabrication methods (ultra low attachment plate: 920 μm; hanging drop: 979 μm), but the hypoxia signal output is 6.5 times higher when fabrication is performed in ultra low attachment plates. It is widely accepted that spheroid size greatly influences *in situ* conditions and final cellular properties. For years scientists have been trying to estimate the critical sizes of various *in vitro* 3D constructs. Indeed, for cancer cellular aggregates it is believed that constructs with diameter between 200 and 500 μm already demonstrate gradients and exhibit oxygen limitation which leads to the necrosis of the core cells (Hirschhaeuser et al., 2010). Some attempts were earlier made to measure or model hypoxic core in MSCs spheroids, leading to controversial results demonstrating that in some spheroids with 100 μm diameter hypoxia was detected (Zhang et al., 2012), and in other experiments no hypoxia occurred in much larger spheroids up to 500 μm (Murphy et al., 2017a). Interestingly, these findings are subsequent with the outcome of our study, since Zhang et al. (2012) formed their spheroids in low attachment plates, while Murphy et al. (2017a) applied the hanging drop method. Therefore, it is questionable if a direct relation of spheroid diameter and hypoxic response in cell spheroids can be made. As presented, additionally to the cell number and spheroid size, the fabrication platform hugely influences the onset of hypoxia.

At the final step, hypoxia reporter cells were applied on microstructured wells to create high numbers of uniform spheroids as advertised by the manufacturer. By using this platform we could demonstrate that if smaller cell numbers were used to create spheroids, no hypoxia occurred in entire plate (20 × 10^4^ cells/ well, 270 cells per spheroid, spheroid diameter of 100 μm). However, we were able to monitor a gradient in hypoxia signal over the microwells, if higher cell numbers were used (100 × 10^4^ cells/ well, 1,350 cells per spheroid, spheroid diameter of 180 μm). Keeping in mind that over 300 genes are up- or down-regulated by HIF-1α, the resulting spheroids represent biologically heterogenic unequal aggregates, with morphologically similar appearance (spheroid sizes). *In situ* oxygen concentration measurements also confirmed unequal oxygen distribution with decreasing concentrations at the well edge.

Taken together, the results of this study demonstrate the influence of cell number and fabrication platform on the onset of hypoxia in hAD-MSCs spheroids. We demonstrated different thresholds for hypoxia when different fabrication platforms are used. The presence of hypoxia, however, has no necessarily negative effect on the cells. As mentioned above, one of the reasons of improved curative potential of MSCs cultivated in 3D could be the increased stromal function as a result of hypoxia (Potapova et al., 2007). Thus, in further experiments, cytokine expression from the spheroids of the same sizes created by different methods must be studied and influence of onset of hypoxia on expression profile must be evaluated. In the future, various 3D fluorescence monitoring techniques must be also applied to better understand spatial distribution of fluorescent signal inside of spheroids. Additional important platforms, where a possible hypoxic response can be studied with the help of reporter MSCs, are advanced 3D co-culture systems. Indeed, different cell types (e.g., cancer cells, endothelial cells) have different oxygen consumption rates and metabolic activities, which can lead to variations in hypoxia onset in spheroids of the same size/cell number. Since such co-cultures are widely used in basic research and drug screening, direct monitoring of hypoxia in these systems will allow a better understanding of cell behavior. If hypoxia must be avoided, spheroids up to 7.5 × 10^4^ cells and 920 μm diameter can be used if hanging drop technique applied, and up to 3 × 10^4^ cells and 600 μm diameter if low attachment plate is used. Nevertheless, using both platforms, non-hypoxic spheroids can be created at the sizes much higher than it was believed before. In microstructured plates also much attention must be paid to the cell numbers used. Moreover, the reason of the radial oxygen gradient from the center to the well edge must be found. The presence of this gradient can also be used to simultaneously study e.g., the influence of different compounds on 3D aggregates under different oxygen tensions.

## Conclusion

In this study we for the first time directly demonstrated critical sizes of hAD-MSCs spheroids in terms of onset of hypoxia. We showed that not only cell numbers, but also spheroid fabrication platforms play a crucial role in the onset of hypoxia. Hypoxia-reporter MSCs detected HIF-1α stabilization in spheroids with 3 × 10^4^ cells (600 μm) if ultra low attachment plates are used. By hanging drop technique, hypoxia can be first detected by spheroids with 7.5 × 10^4^ cells (920 μm). In microstructured plates spheroids with 270 cells and 100 μm did not demonstrate hypoxia, but increased cell numbers (1,350 cells per spheroid and diameter of 180 μm) led to a radial oxygen gradient in the wells. This study underlines, that not only cell type, passage or spheroid diameter are crucial parameters for 3D cell aggregate research, but also the actual fabrication platform critically influences the final spheroid state. Uniform cultivation and spheroid formation platforms would help to create comparable data through different research group and increase the amount of utilizable information for 3D cell aggregate applications. Moreover, hypoxia reporter cells proved to be as an easy and reliable tool to monitor hypoxic response in such systems.

## Data Availability Statement

The raw data supporting the conclusions of this article will be made available by the authors, without undue reservation.

## Ethics Statement

All patients provided their informed consent, as approved by the Institutional Review Board (Hannover Medical School) with the reference number 3475-2017.

## Author Contributions

CS performed most of the experiments and drafted the manuscript. TI generated and analyzed part of the microphotographs and time lapse images. EP and VB provided sensor sequence, introduced sensor sequence into lentiviral vector, helped to analyze and interpretate the results, and proofread the manuscript. AL concepted the manuscript, performed oxygen measurements, participated in the data analysis and results interpretation the results, and proofread the manuscript. All authors contributed to the article and approved the submitted version.

## Conflict of Interest

The authors declare that the research was conducted in the absence of any commercial or financial relationships that could be construed as a potential conflict of interest.

## Supplementary Material

Supplementary Figure 1 Flow cytometry analysis of surface antigens expressed by reporter MSCs (P9) after cultivation in 2D and prior to spheroid fabrication. Cells were stained with respective antibodies conjugated to fluorescent reporters. MSC typical antigens as CD 73 and CD 105 were expressed and cells were tested negatively for hematopoietic (CD34), leucocytic (CD45), and endothelial (CD31) markers.
