# Corrigendum: Hypoxia Onset in Mesenchymal Stem Cell Spheroids: Monitoring With Hypoxia Reporter Cells

**DOI:** 10.3389/fbioe.2022.954422

**Published:** 2022-07-04

**Authors:** Carola Schmitz, Ekaterina Potekhina, Teresa Irianto, Vsevolod V. Belousov, Antonina Lavrentieva

**Affiliations:** ^1^ Institute of Technical Chemistry, Gottfried Wilhelm Leibniz University Hannover, Hannover, Germany; ^2^ Department of Metabolism and Redox Biology, Shemyakin-Ovchinnikov Institute of Bioorganic Chemistry, Moscow, Russia; ^3^ Center for Precision Genome Editing and Genetic Technologies for Biomedicine, Pirogov Russian National Research Medical University, Moscow, Russia; ^4^ Federal Center of Brain Research and Neurotechnologies, Federal Biomedical Agency, Moscow, Russia

**Keywords:** hypoxia sensor, cell spheroids, adipose tissue-derived mesenchymal stem cells, hypoxia reporter cells, oxygen concentration measurements

In the published article, there was an error in the author list. Author Teresa Irianto was erroneously excluded from the article. The corrected **author list** and **Author Contributions** statement appear below.

“Carola Schmitz^1^, Ekaterina Potekhina^2^, Teresa Irianto^1^, Vsevolod V. Belousov^3,4^, Antonina Lavrentieva*^1^”.

