# Supplementary figures and images for: Hypoxia Onset in Mesenchymal Stem Cell Spheroids: Monitoring With Hypoxia Reporter Cells
Source: Front Bioeng Biotechnol. 2021 Feb 5;9:611837. doi: 10.3389/fbioe.2021.611837 (PMC7892969; doi:10.3389/fbioe.2021.611837)

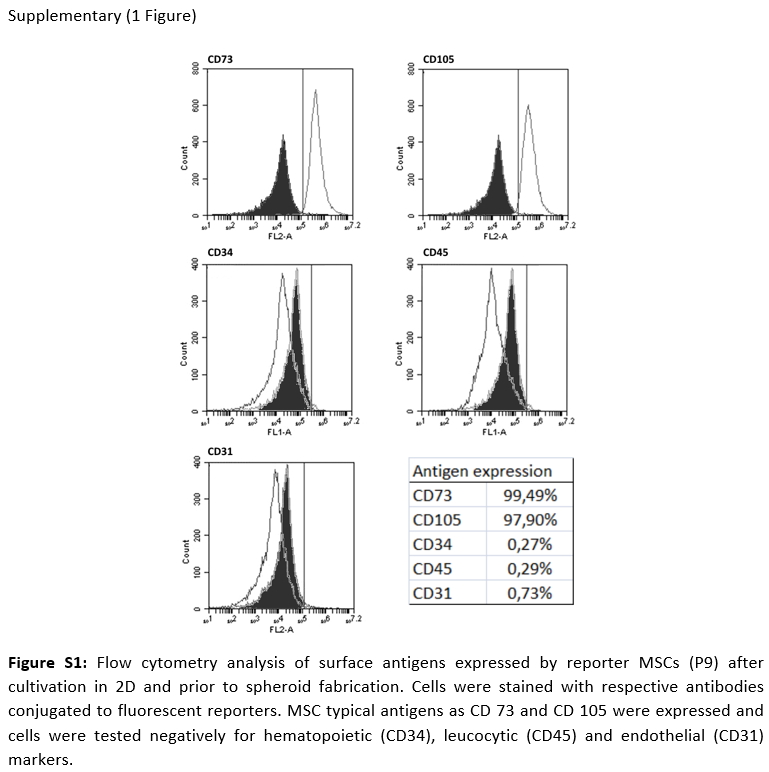

Supplement: Supplementary Figure 1 — Flow cytometry analysis of surface antigens expressed by reporter MSCs (P9) after cultivation in 2D and prior to spheroid fabrication. Cells were stained with respective antibodies conjugated to fluorescent reporters. MSC typical antigens as CD 73 and CD 105 were expressed and cells were tested negatively for hematopoietic (CD34), leucocytic (CD45), and endothelial (CD31) markers. [file Image_1.JPEG]
